# Temporal Expression-based Analysis of Metabolism

**DOI:** 10.1371/journal.pcbi.1002781

**Published:** 2012-11-29

**Authors:** Sara B. Collins, Ed Reznik, Daniel Segrè

**Affiliations:** 1 Program in Bioinformatics, Boston University, Boston, Massachusetts, United States of America; 2 Department of Biomedical Engineering, Boston University, Boston, Massachusetts, United States of America; 3 Center for Biodynamics, Boston University, Boston, Massachusetts, United States of America; 4 Department of Biology, Boston University, Boston, Massachusetts, United States of America; University of Virginia, United States of America

## Abstract

Metabolic flux is frequently rerouted through cellular metabolism in response to dynamic changes in the intra- and extra-cellular environment. Capturing the mechanisms underlying these metabolic transitions in quantitative and predictive models is a prominent challenge in systems biology. Progress in this regard has been made by integrating high-throughput gene expression data into genome-scale stoichiometric models of metabolism. Here, we extend previous approaches to perform a Temporal Expression-based Analysis of Metabolism (TEAM). We apply TEAM to understanding the complex metabolic dynamics of the respiratorily versatile bacterium *Shewanella oneidensis* grown under aerobic, lactate-limited conditions. TEAM predicts temporal metabolic flux distributions using time-series gene expression data. Increased predictive power is achieved by supplementing these data with a large reference compendium of gene expression, which allows us to take into account the unique character of the distribution of expression of each individual gene. We further propose a straightforward method for studying the sensitivity of TEAM to changes in its fundamental free threshold parameter *θ*, and reveal that discrete zones of distinct metabolic behavior arise as this parameter is changed. By comparing the qualitative characteristics of these zones to additional experimental data, we are able to constrain the range of *θ* to a small, well-defined interval. In parallel, the sensitivity analysis reveals the inherently difficult nature of dynamic metabolic flux modeling: small errors early in the simulation propagate to relatively large changes later in the simulation. We expect that handling such “history-dependent” sensitivities will be a major challenge in the future development of dynamic metabolic-modeling techniques.

## Author Summary

Understanding the dynamic response of microorganisms to environmental changes is a major challenge in systems biology. In many cases, these responses manifest themselves through changes in gene transcription, which then propagate to adjust flow through metabolism. Here, we implement a Temporal Expression-based Analysis of Metabolism (TEAM) by dynamically integrating a genome-scale model of the metabolism of *S. oneidensis* with high-throughput measurements of gene expression and growth data. TEAM recapitulates the complex cascade of secretion and re-uptake of intermediary carbon sources that *S. oneidensis* exhibits in the experimental data. We show that these complicated metabolic behaviors are best captured when TEAM explicitly accounts for each gene's unique transcriptional signature. Furthermore, by way of a newly proposed sensitivity analysis, we reveal and study the inherent difficulty of dynamic metabolic flux modeling: small changes early in a simulation can easily spread and lead to significant changes towards the end of it. We expect that further development of robust dynamic flux balance methods will need to overcome such “history-dependent” sensitivities in order to achieve increased predictive accuracy.

## Introduction

In response to environmental changes, microbes modulate their metabolic activity through a complex interplay of biochemical and regulatory networks. The dynamics of these changes is a poorly understood process, relevant for many applications ranging from infectious diseases to environmental remediation. With the rise of genome-scale stoichiometric models of metabolism [1], these challenges have been addressed through the development of algorithms that overlay gene expression data onto these models to quantitatively study the effects of genetic regulation on cellular metabolism. One of the most widely used approaches for genome-scale predictions of metabolic fluxes is Flux Balance Analysis (FBA) [2]–[5]. FBA uses a steady state approximation and linear programming to determine optimal solutions to the problem of allocation of metabolic resources through a metabolic network. Several FBA-based methods have been proposed to integrate measurements of mRNA abundance, often with the goal of improving the prediction of fluxes in a metabolic network. One general approach is to constrain the maximum flux through reactions whose catalyzing enzyme genes have low expression levels. Some examples of this strategy include regulatory FBA (rFBA) [6], steady-state regulatory FBA (SR-FBA) [7], integrative FBA (iFBA) [8], E-Flux [9] and Probabilistic Regulation Of Metabolism (PROM) [10]. Another way to integrate context-specific data is to match changes in flux with statistically significant changes in mRNA levels over time, a strategy employed by Metabolic Adjustment by Differential Expression (MADE) [11]. Yet another strategy is not to constrain fluxes directly, but instead to penalize reactions whose fluxes deviate from their coding genes' expression by introducing a cost function to be minimized. Two examples of this strategy include that of Shlomi *et al.* [12] and Gene Inactivity Moderated by Metabolism and Expression (GIMME) [13]. GIMME, the method upon which we will build in this work, is a particular extension of FBA that maximizes metabolic consistency with gene expression data, producing a set of fluxes that both satisfy the stoichiometric constraints of the metabolic model and provide a context-specific prediction that is informed by experimental data.

These methods vary widely in the details of their implementation, but they all ultimately have to grapple with a number of common obstacles and limitations [14]. Most notably, irrespective of whether regulatory information is used as a constraint or as part of the objective, these approaches require making some assumptions on how mRNA expression levels end up affecting fluxes. Part of the problem is the complex relationship between mRNA expression and protein levels [15]. In this respect, methods that use expression levels as part of the objective (e.g., through maximization of consistency) rather than as hard constraints, have the advantage of allowing a certain flexibility in the final choice of flux values. An additional issue associated with the use of mRNA levels to inform fluxes is the necessity (in many but not all approaches) to choose a universal threshold below which expression can be effectively deemed unlikely to support flux. It is also important to note that most of the literature on integration of expression with flux balance modeling focuses on static cases, without exploring the feasibility and potential issues associated with the application to time course data.

**Figure 1:**
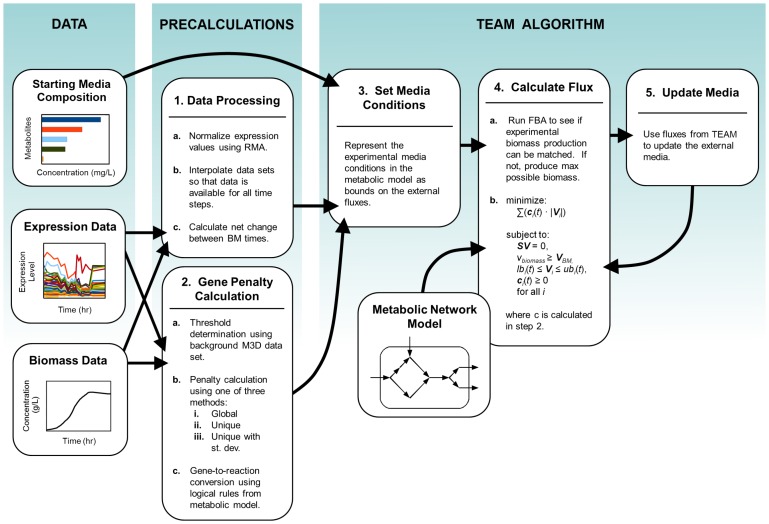
Workflow for integrating multiple data types with TEAM. TEAM integrates three types of experimental data: starting media composition, expression data, and biomass data. Pre-calculations include normalization of the gene expression data, interpolation of all data sets, and calculation of gene penalties based on the gene expression data. For a given time interval, TEAM calculates the metabolic flux distribution most consistent with gene expression and biomass data. It applies this result to update media conditions for the subsequent time interval.

Here, in an attempt to advance our understanding of the interplay between metabolism and regulation in time-dependent processes, we present a new algorithm named TEAM (Temporal Expression-based Analysis of Metabolism). TEAM integrates dynamic flux balance analysis (dFBA) [16] with time-dependent gene expression data, using a cost minimization scheme similar to GIMME [13]. In addition to representing a unique example of integration of time-dependent gene expression with dFBA, the TEAM approach introduces some important innovations relative to the GIMME method. In particular TEAM takes advantage of an additional large compendium of gene expression data [17] to estimate gene-specific expression penalties, effectively taking into account the individuality of expression patterns identifiable in different genes. Furthermore, through TEAM, we introduce a new, simple sensitivity analysis that helps estimate the predictive power of the approach under different choices of parameters. For a succinct overview of the TEAM algorithm, refer to **Figure 1**.

To test TEAM's ability to predict bacterial behavior in the face of changing environmental conditions, we apply it to data collected during batch growth of *Shewanella oneidensis* MR-1 under minimal lactate aerobic conditions [18]. *S. oneidensis* is a dissimilatory metal-reducing gammaproteobacterium that was discovered in Lake Oneida, NY in 1988 and has since been shown to be able to utilize over 20 different electron acceptors [19]–[21]. This unusual ability allows *Shewanella* species to adapt to many different habitats that often contain oxic/anoxic transition zones [19] and an abundance of various fermentation products such as lactate, formate and hydrogen [22]. In the experiment we use for our analysis, extracellular metabolites (high performance liquid chromatography, HPLC), gene expression levels (Affymetrix microarrays), and population size (optical density, OD) were measured over the course of 50 hours (see also Materials and Methods). Similar to previous *S. oneidensis* growth experiments [23], [24], these data displayed excretion and re-uptake of acetate and pyruvate during growth, a pattern that could not be explained by regular dFBA simulations [18]. Eiteman *et al.* attribute this phenomenon, called overflow metabolism, to the imbalance between the enzymatic capacity of the TCA cycle to fully oxidize acetyl-CoA and the rate of carbon consumption [25]. The excess production of NADH by the TCA cycle is thought to repress the TCA cycle genes themselves, forcing the usage of anaerobic pathways that do not produce NADH, such as the acetate generation pathway. One of the goals we set in developing TEAM is precisely to be able to reconcile gene expression data with metabolic constraints, to help understand otherwise indecipherable metabolic behavior, such as the metabolic overflow observed in the HPLC data. More generally, we propose TEAM as a strategy for marrying two often detached views of bacterial physiology: environmental resource utilization and internal enzymatic functional states. The complexity that arises when these two views are integrated allows one to draw important conclusions about the behavior of bacteria that may not have been previously possible.

## Results

### Comparing TEAM with dFBA

**Figure 2:**
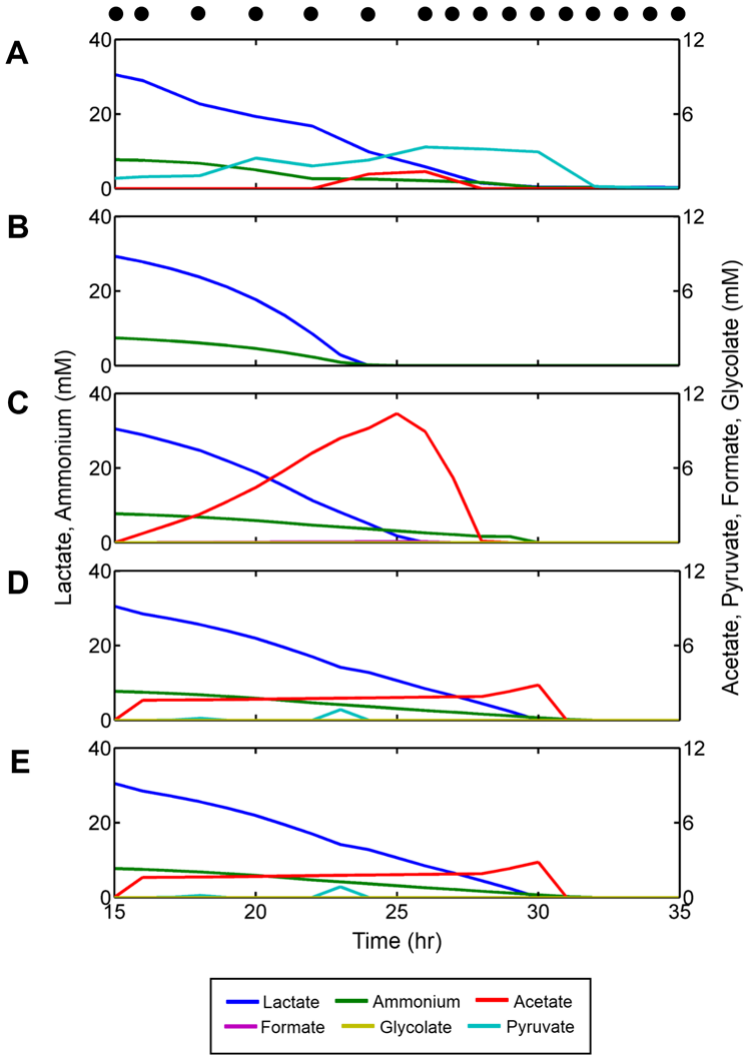
A comparison of results across different methods for a representative penalty threshold. The media contained 36 mM L-Lactate, 13 mM D-lactate, 9 mM ammonium, and other minimally required nutrients. The oxygen concentration was set to 10 mM at each time point, mimicking the controlled 100% dissolved oxygen (DO) concentration from the experiment. The resulting usage dynamics of several metabolites of interest (including combined DL-lactate, ammonium, pyruvate, acetate, formate and glycolate) as predicted by dFBA are compared to experimental data. (A) HPLC Data, (B) dFBA, (C) TEAM with a global penalty threshold (Type 1), (D) TEAM with a gene-specific penalty threshold (Type 2), (E) TEAM with a gene-specific penalty threshold normalized by standard deviation (Type 3). Black dots represent hours when microarray measurements were taken.

Our interest in exploring novel avenues for integrating dynamic models of metabolism with measurements of gene expression was partially motivated by the desire to account for metabolic behaviors that could not be predicted through regular dFBA, such as the overflow metabolism in *S. oneidensis* described above. Specifically, we wanted to recapitulate three striking features of the experiment as observed from the HPLC data: (1) the nearly simultaneous exhaustion of all carbon sources and ammonium in the media, (2) the excretion and subsequent re-uptake of acetate from the media, and (3) the excretion and subsequent re-uptake of pyruvate. These experimental measurements are shown in **Figure 2A**. The results of a standard dFBA simulation for this system are shown in **Figure 2B** (see Materials and Methods for details). Using conventional dFBA we were able to qualitatively match the predicted lactate and ammonium dynamics to those in the collected data. However, the method failed to predict the correct depletion times for both lactate and ammonium, and also failed to account for the presence of acetate and pyruvate.

The failure of dFBA to capture some unique features of our experiment led us to try to incorporate gene expression into our simulation. We did so by merging GIMME with dFBA into a preliminary version of TEAM. Each iteration of TEAM completes a GIMME optimization, mathematically formulated, in analogy to other FBA algorithms ([2]) (see Materials and Methods for details) as:
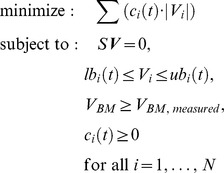
where *S* is the stoichiometric matrix, *lb_i_* and *ub_i_* are, respectively, the lower and upper bounds of flux *V_i_*, *V_BM_* is the biomass production rate (or growth flux) and *c_i_* is a penalty assigned to reaction *i* based on the expression of its constituent genes.

TEAM's implementation of GIMME diverges from the original implementation in [13] in two ways. First, TEAM uses experimental measurements of biologically necessary fluxes, most notably the growth rate (or, potentially, of any exchange flux) to impose specific magnitudes to the corresponding fluxes in the model. This is in contrast to [13], where the minimal flux passing through each required metabolic functionality (RMF) was calculated as some percentage (a free parameter in the system) of the maximal flux which could pass through that RMF (as calculated using flux balance analysis, see Materials and Methods).

Second, and most importantly, TEAM and GIMME differ in how the coefficients *c_i_* of the penalty function are calculated. This penalty was calculated in [13] by first propagating expression measurements from each annotated gene in the model to its corresponding reaction using the Boolean gene-to-reaction mapping rules. Then, if the expression associated with a reaction exceeded a user-defined threshold *p*, that reaction was assigned a penalty *c_i_* of zero. Otherwise, the reaction was assigned a penalty equal to the difference between the threshold and its expression. In TEAM, we modified this protocol by first calculating the penalty of each gene, and then propagating this penalty up to each reaction. In the following section, we will show how this small change enabled us to incorporate more data on the expression characteristics of each gene into TEAM to generate markedly better predictions.

As a first trial, we assigned a common, global penalty threshold (herein referred to as a Type 1 threshold) to all genes in the model. We tested many thresholds, and found that the threshold falling in the *θ = *70^th^ percentile of all expression measurements from the microarrays in our experiment appeared to give the most accurate predictions. We will defer from commenting on the quantitative accuracy of this version of TEAM until later on, when we will do so not only across all possible penalty thresholds, but also across different methods of assigning such thresholds.

As shown in **Figure 2C**, the TEAM simulation with Type 1 penalty threshold was able to reasonably capture the qualitative dynamics of acetate in addition to lactate and ammonium. Although the magnitude of TEAM's predicted acetate dynamics greatly overestimated the experimental data, the results were nevertheless promising. The predicted acetate dynamics showed significant improvement over the dFBA simulation, which exhibited no acetate dynamics whatsoever. However, despite testing of many penalty thresholds, we were not able to find any TEAM simulations with Type 1 thresholding which displayed any pyruvate secretion/uptake. This, combined with a great deal of variability in the timing and magnitude of acetate dynamics, prompted us to search for ways to refine TEAM.

### Gene Individuality

**Figure 3:**
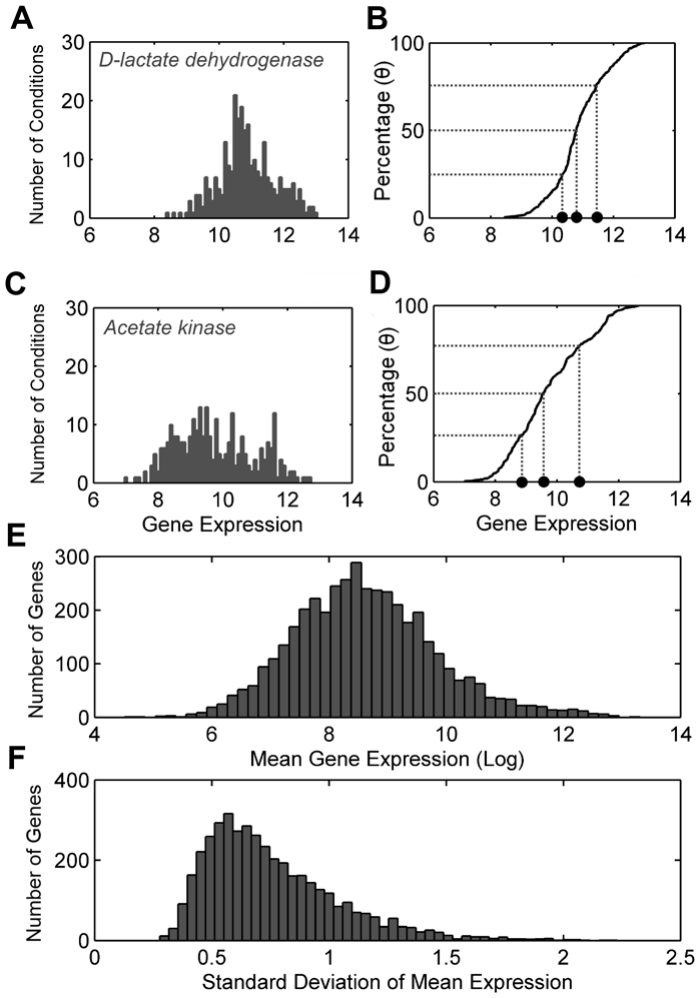
Overall distribution of *S. oneidensis* gene expression measurements with two individual genes highlighted. Distributions calculated using a pooled set of data from M3D [17] and time-course experimental data [18]. For both example genes, the distribution of gene expression measurements and the corresponding cumulative distribution function (CDF) are shown. For each CDF, individual gene penalty thresholds are found for common percentiles *θ* = 25%, 50% and 75%. D-lactate dehydrogenase expression measurements (A) have a higher mean expression and a more pronounced peak than acetate kinase (C), which is more uniformly distributed. The corresponding CDFs capture this variation in distribution. D-lactate dehydrogenase expression penalties (B) are higher and less distributed than those of acetate kinase (D). Mean gene expression (E) and standard deviation (F) over all genes for a single time point are also shown. All microarrays contain 4230 gene products, and each individual distribution contains 310 data points; 19 come from the experimental time-course and 281 from M3D.

Despite our success in using TEAM to recover acetate dynamics, we were still unable to capture the dynamics of pyruvate in the media. We began to consider the possibility that assigning an identical penalty threshold to each gene in the model was causing us to lose valuable information regarding the likelihood that each gene was active. This motivated us to inspect the distribution of gene expression values for each gene in the *S. oneidensis* model. We assembled a compendium of gene expression data for *S. oneidensis* using the M3D database [17]. For each gene in the database, we generated a histogram of gene expression values built from all the available microarrays in the database and supplemented with our own microarrays from the current experiment. A representative sampling of these gene expression histograms is shown in **Figures 3A and 3C**.

It became quite clear that two genes in the model could have significantly different expression characteristics. This is illustrated in **Figures 3A–3D**, which show the distribution of expression measurements for two enzymes essential to lactate metabolism in *S. oneidensis*. The distribution of measured expression for D-lactate dehydrogenase was found to be tightly centered around its mean with a very small standard deviation. In contrast, expression for acetate kinase exhibited a much broader multi-modal distribution with a significantly higher standard deviation. This variability in the distribution of expression values became even more striking when plotting the distribution of the means and standard deviations of expression measurements across all genes, shown in **Figures 3E and 3F**, respectively. A gap of over two orders of magnitude was observed over all genes in the model. Biologically, the disparities in expression signatures among the genes in *S. oneidensis* may have arisen from a variety of biological sources. One possibility is that some genes may code for mRNAs with relatively high translational efficiency, or for enzymes with relatively high catalytic rates, thus requiring fewer mRNAs in order to achieve an identical metabolic flux. Another possibility is that the products of some genes may be constantly required for the operation of the cell (such as the enzymes of central carbon metabolism), while others are only needed in particular situations (such as transporters for specific carbon sources).

Prompted by the observation that individual genes showed unique expression characteristics, we developed two new methods for calculating penalty thresholds customized to each gene in the model. In the first (referred to herein as Type 2), we used our compendium of gene expression data to calculate a cumulative distribution function (CDF) for each gene in the metabolic model. Then, a common percentile *θ* was chosen for all genes in the model. Next, we used each gene's CDF to assign the expression level corresponding to this percentile as the penalty for that particular gene. We then calculated a penalty for each and propagated this penalty to each reaction in the model as described earlier and in the Materials and Methods. The second new thresholding method (Type 3) proceeds exactly as Type 2 thresholding, except that each gene's penalty is now normalized by that gene's standard deviation, as calculated from our compendium of expression data. Upon completing TEAM simulations with these two new thresholding methods for the same *θ* as the most accurate Type 1 simulation, we immediately observed the appearance of excretion and subsequent re-uptake of pyruvate in the media (**Figures 2D, 2E**).

**Figure 4:**
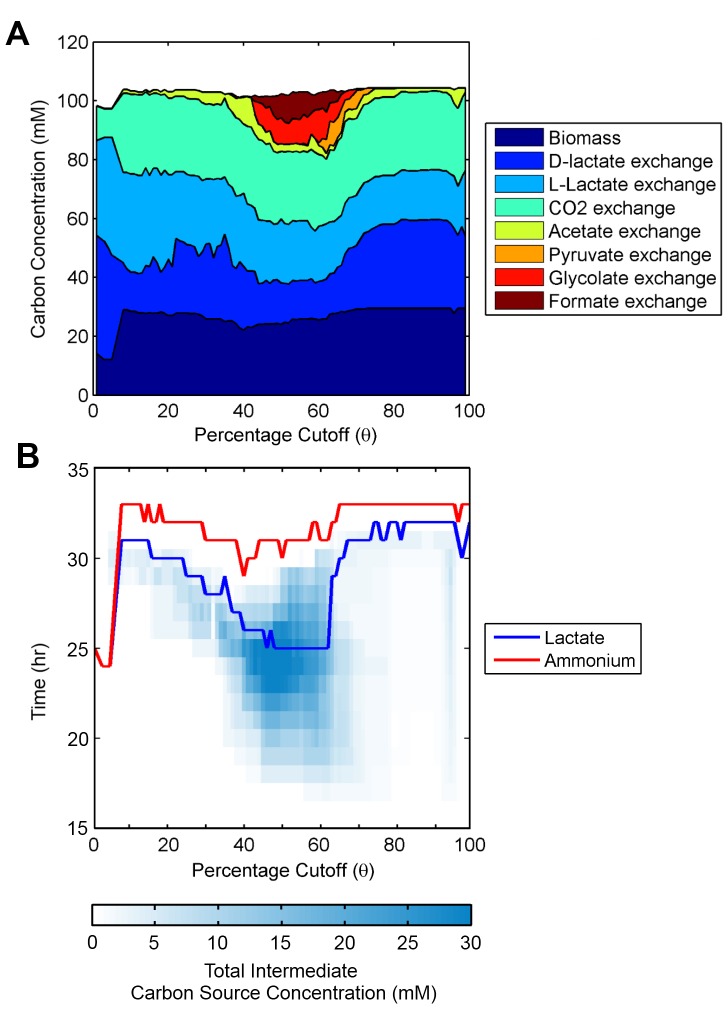
Sensitivity analysis for Type 2 gene-specific threshold. (A) Total carbon concentration in media for each penalty threshold *θ*, summed over all time points. Penalty thresholds between 40% and 75% exhibit enrichment for intermediate carbon sources formate, glycolate, pyruvate. (B) Extinction time of lactate and ammonium in the media. Lactate runs out significantly earlier for intermediate penalty thresholds. Heatmap indicates the total media concentration of secreted carbon sources (acetate, pyruvate, glycolate, formate).

Next, we sought to systematically assess whether our refined penalty methods show significantly improved predictive capabilities when compared to dFBA and TEAM with Type 1 thresholding. We pursued this in two ways. First, we studied the predicted secretion patterns of all of the TEAM methods across the entire range of potential penalty thresholds *θ*. To do so, we calculated the total amount of each different carbon source found in the media over the entire course of the simulation for each penalty threshold. We did this for each penalty threshold and for all three TEAM methods. The results are shown in **Figures 4**, **S1A**, and **S2A**. These figures highlight that only the Type 2 and 3 TEAM methods with unique penalties for each gene were able to predict the excretion and re-uptake of pyruvate. The Type 1 method failed to predict any pyruvate dynamics in the external media for the entire range of possible penalties. Despite predicting pyruvate, in a small range of penalty thresholds the Type 2 and 3 methods also spuriously predicted the excretion and re-uptake of formate and glycolate, two intermediary metabolites which we confirmed were not present in the experiment.

**Figure 5:**
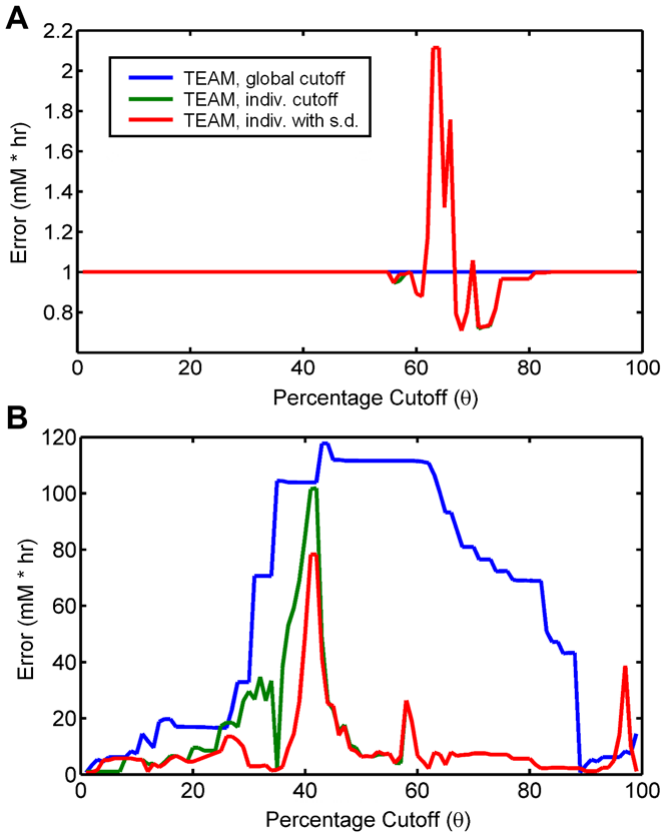
A measure of predictive accuracy between pyruvate and acetate excretion behavior. For all percentage thresholds *θ* between 1% and 99%, the quality of predictions for (A) pyruvate and (B) acetate secretion and utilization behavior was calculated using the residual sum of squares between the experimental HPLC measurements and the model predictions for all three gene penalty calculation types. Only the Type 2 and 3 penalty thresholds predict the secretion of pyruvate, occurring between *θ = *55% and *θ = *82%.

As a second step towards assessing the effect of different thresholding methods on secretion patterns, we developed a quantitative assessment of their relative predictive accuracy. We decided that because we were most concerned with recapitulating the excretion and re-uptake of pyruvate and acetate, we would focus on each method's ability to accurately predict the dynamics of these metabolites. For a given simulation, we calculated the residual squared error between the predicted concentration of acetate and pyruvate in the media and summed over all time points. The total error was plotted against penalty threshold for all three TEAM methods and shown in **Figure 5**. The results illustrate that by accounting for the individuality of genes, the two refined TEAM methods performed at least as well or better than the original method for all penalty thresholds. For penalty thresholds in the range of 30% to 70%, the refined methods perform significantly better, while at either extreme of the thresholds, the difference between methods is smaller. For all three TEAM methods, we found that changing the penalty threshold had a large impact on the quantitative accuracy of our model, and enabled us to make an informed choice of a penalty threshold which seemed to best match our experimental observations.

Our promising results using custom thresholds with *S. oneidensis* prompted us to test whether accounting for the heterogeneity of gene expression would also facilitate the integration of gene expression in flux balance models across other datasets. Specifically, we tested TEAM on a yeast growth transition dataset [26], previously used to evaluate the performance of the MADE approach [11], as well as on experimental data on the behavior of a synchronized yeast population undergoing metabolic oscillations [27]. In both cases we found that gene-specific thresholds (Type 2) improve the consistency of flux predictions with gene expression data (**Figures S4 and S5B**), as well as the capacity to predict metabolite secretion (**Figure S5A**).

### Examining Internal Fluxes in TEAM

Given that our refined penalization methods (Types 2 and 3) produced quantitatively more accurate results than the original (Type 1) method, we next inspected how varying the penalty threshold for these refined methods influenced the predicted dynamics of pyruvate, acetate, glycolate, and formate secretion. As shown in **Figure 4**, as the Type 2 and Type 3 penalty thresholds increase, zones of qualitatively different behavior emerge. Acetate is always excreted regardless of the penalty threshold (**Figure 4A**). Glycolate, formate and pyruvate, however, are only excreted in the intermediate zone between penalty thresholds *θ* = 45% and *θ* = 72%. Furthermore, as shown in **Figure 4B**, in this intermediate zone, lactate is completely consumed within 28 to 30 hours, while in the peripheral zones it is consumed between 30 and 34 hours. In this intermediate zone, we find that the early exhaustion of lactate is strongly correlated to high concentrations of intermediate carbon sources (pyruvate, acetate, formate, and glycolate) in the media.

In a very narrow range of thresholds, from *θ* = 65% to *θ* = 72%, we observe the secretion of acetate and pyruvate, but not glycolate and formate. This qualitative agreement led us to identify this range of thresholds as the “optimal range” within which we expected TEAM's predictions of metabolic activity to be most accurate. However, because we did not obtain any measurements of internal fluxes from the experiment, we were unable to further explore how these predictions correlated with *in vivo* fluxes. Instead, we turned to studying TEAM's novel predictions of formate and glycolate secretion. Although these two metabolites were not observed in the HPLC measurements, their appearance in TEAM's predictions suggests that *S. oneidensis* may be capable of secreting the two metabolites under some as-yet unidentified conditions.

**Figure 6:**
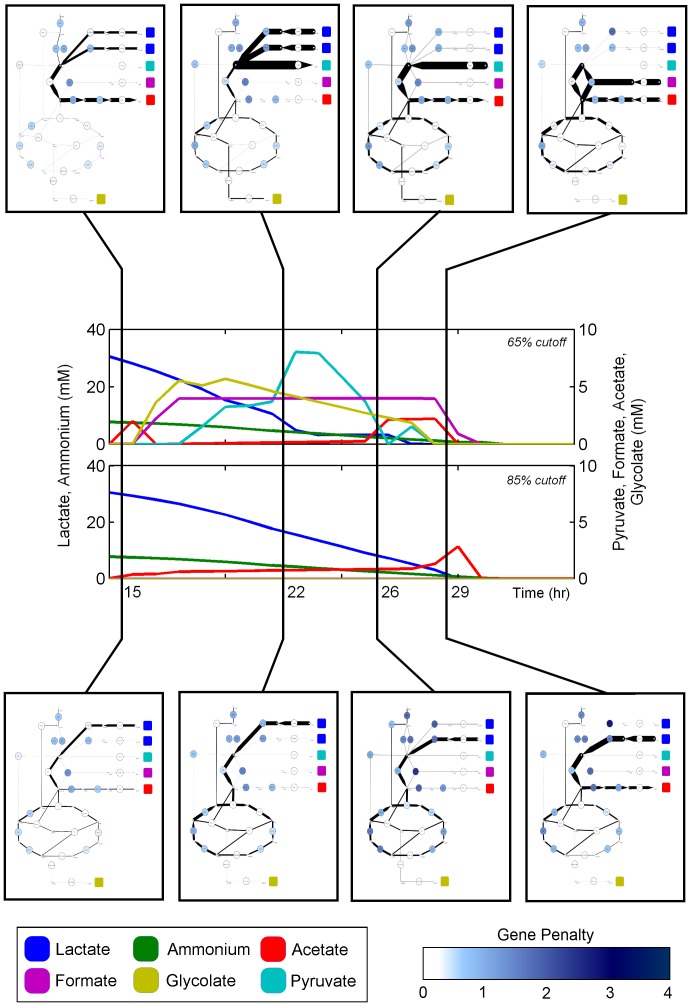
A comparison of internal flux profiles for two different penalty thresholds. Superposition of metabolic flux onto central carbon metabolism of *S. oneidensis*. Top panel corresponds to a Type 2 penalty threshold of 65%, and bottom panel to a Type 2 penalty threshold of 85%. Large nodes and edges on the networks represent reactions and small nodes correspond to metabolites. The colors of the large nodes correspond to the penalty associated with that reaction. Colored squares on the network plots identify the transport reactions for each exchange metabolite. A network key and reaction and metabolite details can be found in Figure S3, Table S1 and Table S2 respectively. Detailed time-course flux predictions are provided in Supplementary Dataset S1.

We decided to investigate in fine detail the mechanisms linking the secretion of formate and glycolate to lactate exhaustion. This scenario is analyzed in **Figure 6**, which highlights, at the individual flux level, several of the dramatic differences in TEAM predictions as the penalty threshold is increased. In one of the three qualitatively different behaviors observed (at a threshold of 65%), lactate is imported significantly faster than the measured rate in HPLC. This excess of imported carbon is then funneled through several pathways including the TCA cycle, the glyoxylate shunt, a formate-producing cycle, and acetyl-CoA synthetase. Each of these pathways results in the production of carbon compounds (CO_2_, glycolate, and acetate, respectively) which are excreted into the media. In contrast, the simulation at a threshold of 85% displays a more tempered rate of lactate usage, leading, through the TCA cycle, to secretion of CO_2_ and acetate.

Given that our algorithm minimizes the sum of the absolute values of fluxes, it is somehow surprising that, in the 65% threshold regime, TEAM would predict overflow metabolism. Can this be explained in terms of actual energetic requirements for the cell? We found that the increase in NADH produced as a result of importing excess lactate and metabolizing it via lactate dehydrogenase (which produces pyruvate and NADH from lactate and NAD^+^) provided adequate reducing power to the cell. The resulting pyruvate is then converted into whichever intermediate carbon sources (acetate, formate, or glycolate) minimize the inconsistency between gene expression and flux. Previous studies using isotope tracing to infer flux have reported similar increased activity of both the glyxoylate shunt and a proposed serine oxidation cycle producing formate in *S. oneidensis* in aerobic, carbon-limited conditions [23]. Here, our simulations suggest that the transcriptional response of *S. oneidensis* to changing environmental conditions dictates the routing of flux into these pathways.

Our investigation of pyruvate dynamics led us to another curious but intuitive observation: we found that the availability of a large repertoire of intermediate metabolites early in the time course led to a high diversity of metabolic activity later on in the simulation. Because these metabolites can be funneled through a larger variety of pathways than lactate, the model is able to select from among all these pathways to find the minimally penalized reaction path. For example, for several hours in the top panel of **Figure 6**, TEAM predicts that both glycolate and formate are secreted into the media. This means that later on, TEAM has the option of importing either one of these carbon sources, but actually imports glycolate first and then formate. This is a direct result of a high penalty associated with pyruvate formate lyase required to utilize formate and no penalty associated with the reactions required to import glycolate. Thus, the model chooses the sequence of carbon source usage in best agreement with the gene expression. In contrast, for a higher penalty threshold in the bottom panel of **Figure 6**, TEAM has no access to formate and glycolate in the media. This means that while the gene expression is identical to the intermediate zone, a different set of environmental conditions results in starkly different behavior.

## Discussion

The growing abundance of high throughput gene expression datasets has led to a call for methods integrating these experimental data with stoichiometrically based genome-scale models of metabolism. Our implementation of TEAM explored some of the challenges associated with developing such methods. In particular, we found useful ways of incorporating assorted data types (OD, microarray data) to constrain some of the otherwise free parameters of TEAM. We discovered that accounting for the heterogeneity of expression across different genes leads to an increase in predictive accuracy. Most importantly, it was simple to identify those penalty thresholds expected to be the most accurate, simply by matching qualitative predictions (i.e. acetate and pyruvate secretion) to experimental observations. Despite these successes, we still observed qualitatively broad shifts in TEAM's predictions as certain parameters varied, and we introduced a simple technique for sensitivity analysis which teased out precisely where these shifts took place. This sensitivity analysis enabled us to identify a narrow range of penalty thresholds, within which we were confident of TEAM's predictions. This suggests that in future analyses, it may be more appropriate to report a summary of results across the whole spectrum of thresholds, using a metric of agreement with experimental data as a criterion for choosing the “optimal threshold”. We suggest that such sensitivity analyses should become a central component of future efforts to integrate gene expression with flux balance models.

A common thread that ran through each of our successive improvements to the original GIMME algorithm was the use of experimental measurements to improve the predictive accuracy of TEAM. Rather than use all of our data to evaluate the performance of TEAM, we found that some types of data were better suited to generating more informed models, while others seemed to be more useful in validation. In particular, one user-defined parameter from the original GIMME algorithm (the minimal RMF flux) was completely eliminated simply by linking its value to the observed experimental biomass flux. Another parameter, the penalty threshold of each gene, morphed from a common value for all genes to a quantity unique to each gene and directly determined by prior measurements of that gene's typical expression behavior. The elimination of these otherwise relatively unconstrained parameters enabled us to systematically evaluate the performance of TEAM. Furthermore, these improvements came at very little cost in terms of experimental effort. The collection of OD data is standard in metabolic engineering, and our supplementary microarray data was freely available in the M3D database. Building on prior work on the GIMME algorithm, we assessed TEAM's sensitivity to penalty thresholds and concluded that broad, qualitative changes in TEAM's predictions (such as the appearance of glycolate and formate in the media) were not due to changes in the penalization of a single or small group of genes. Instead, it was the total consistency of fluxes over the entire network that led to these shifts in TEAM's behavior. In many cases, we found two genes in the same pathway in *S. oneidensis* exhibited opposing expression behavior (i.e. one gene's expression would be rising, while the other's would simultaneously fall). By integrating these expression measurements with a model that enforces mass-balance constraints, TEAM was able to reconcile otherwise conflicting signals and output a coherent pattern of metabolic fluxes that best fit the available data. This highlighted the value that methods integrating expression data with metabolic models have over more classical techniques for analyzing expression data in isolation, like simple pathway enrichment. Incompatible trends in the expression of the enzymes of one metabolic pathway were made much more coherent by connecting them to the operation of the metabolic network as a whole.

Looking carefully at our predictions, we found that even the best TEAM predictions did not precisely match the timing and magnitude of acetate and pyruvate dynamics from the experimental data. While there may be many sources for the discrepancies between TEAM's predictions and the data, one prominent and unresolved question regards the error associated with using mRNA abundance as a proxy for the activity of a metabolic reaction (typically related to the total concentration of enzyme in the cell). Recently, a number of experimental studies [15], [28] have addressed the question of correlation between mRNA and protein abundance. While there is some correlation between mRNA and protein levels, it now appears that a more relevant question is the relationship between the half-lives and production rates of both mRNA and protein. In particular, Schwanhäusser *et al.* [15] showed that different genes displayed characteristically different combinations of mRNA and protein half-lives. These combinations were linked to a model of energetic resources in the cell, based on the argument that different blends of mRNA and protein stability may be associated with the functional role a particular protein plays within the cell [15]. Although difficult to obtain, information about protein half-lives could be directly integrated into TEAM by calculating a gene's penalty based on its expression integrated over a time interval. This may lead to delays in the onset of a penalty, as well as penalties that remain active for long periods of time. It is noteworthy that such time-dependent improvements would heavily rely on TEAM's dynamic nature; static simulations of GIMME would be unable to capture the diversity of dynamic behaviors in mRNA and protein. Finally, even the integration of precise proteomics data needs to be treated with care. Fendt *et al.* [29] report cases in which changes in metabolite concentration correlate both positively and negatively with enzyme concentration, suggesting that one should not necessarily expect strong correlations between metabolic flux and enzyme abundance.

Our study of the predicted appearance of glycolate and formate in the media led us to another major conclusion: a spurious prediction about the excretion of metabolites early in a simulation can lead to very significant qualitative errors from TEAM later on. The difficulty was that the gene expression TEAM used was intimately tied to very specific environmental conditions. If TEAM predicted media conditions that included nutrients not found in the true experimental conditions, then the simulation had access to certain metabolic pathways (for example, C1 metabolism of formate) which could not have been active in the experiment. We reasoned that by imposing adequately high conformity with gene expression with high penalties, we would be able to prevent this spurious behavior. In fact, this is precisely what we observed: at high levels of penalty threshold, we no longer found glycolate or formate present in the media. The disappearance of these two metabolites was directly linked to a reduction in the import of lactate early in the simulation. In general, along the time course, there is a tight mutual dependence between the rates of metabolite uptake/secretion, and the transcriptional regulation of the pathways for producing or utilizing those metabolites. For *S. oneidensis*, this amounted to the rapid intake of lactate (faster than required if oxidative phosphorylation were used, but slower than required if glycolate and formate were secreted), resulting in the overflow metabolism associated with the secretion of acetate and pyruvate. We expect that future efforts to develop dynamic genome-scale metabolic models will encounter similar temporal sensitivity issues. Improvements in accuracy will depend on the ability to prevent predictions of qualitatively spurious media conditions.

Finally, the history-dependent sensitivity of TEAM underscores the underappreciated interplay between gene expression and environmental conditions. The upregulation of genes associated with a particular pathway is frequently used as a proxy for inferring increased metabolic activity in the pathway itself, e.g. in the analysis of large expression datasets associated with human disease, such as cancer. Our work with TEAM suggests that the inference of metabolic activity directly from gene expression data can be quite misleading. In addition to effects associated with the delay between transcriptional and metabolic response, distinct extracellular environments, coupled with identical gene expression profiles, can re-organize the activity of metabolic pathways in substantially different ways. Therefore, we would argue that future studies of metabolism must carefully account for the environmental context within which gene expression is measured.

## Materials and Methods

### Data Inputs and Interpolation

TEAM uses four sets of data as inputs, in addition to the stoichiometric model: time-dependent, OD-based, biomass measurements (*OD*), time-dependent gene expression microarray measurements (*EXP*), a reference compendium of gene-expression data unrelated to the current experiment (in our case, obtained from the M3D [17] database and labeled *M3D*) and initial concentration of nutrients in the growth medium (*MEDIA*). To make the data sets *OD, EXP, M3D*, and *MEDIA* congruent with each other and with the algorithm architecture, the data is interpolated for the appropriate time interval across the entire experimental period. We used a time interval Δ*t* of 1 hour, and performed the interpolation using the Matlab *interp1* function.

The reactions in the stoichiometric model can be characterized as either exchange or biological fluxes. Biological fluxes are associated with enzyme-catalyzed metabolic reactions and transport reactions. Exchange fluxes act as source and sink reactions that balance the biological fluxes. Formally we define these two sets as follows:
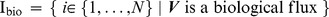
(1)

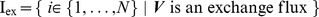
(2)By convention, a positive flux through an exchange reaction means that a metabolite is secreted, and conversely a negative exchange flux corresponds to uptake of a metabolite. Therefore, a lower bound on an exchange flux is equivalent to the maximal uptake rate of the corresponding transportable metabolite in a time interval Δ*t*.

### Implementation of TEAM

TEAM is based on the previously described dFBA [16] and GIMME [13] methods. For a comprehensive overview of the TEAM method, see **Figure 1**. TEAM produces a time-series of metabolic flux predictions ***V***(*t*) by identifying, at each (discrete) time *t*, the metabolic flux distribution that is most consistent with measured gene expression data at that time. The resulting flux distribution is assumed to be valid for a time interval Δ*t*, and is used to update the concentrations of nutrients in the media for the subsequent time interval. In this way, a series of static optimizations are linked to each other by the repeated updating of external media nutrient availability.

A TEAM simulation is initialized by setting the initial concentration of nutrients in the growth medium. Let *e_i_*(t) represent the concentration (in mM, considering a working volume of 1liter) of the *i* ^th^ component of the medium at time *t*. We initialize ***e*** to reproduce the experimentally known initial medium composition at time *t* = 0:
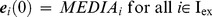
(3)


Next, we initialize the problem so that a dFBA iteration can be completed. We use the current metabolite concentrations to infer the lower bounds (meaning maximal possible inflow) on all exchange fluxes:
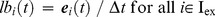
(4)Furthermore, we set the initial biomass concentration in TEAM equal to the appropriately scaled experimentally measured optical density: *BM(0) = OD(0)*.

In order to consistently solve the problem for the total biomass available, at each time point we convert the constraints on the biological fluxes from specific (*lb* ^(0)^, *ub* ^(0)^), defined per unit of biomass, mmol/gDW·hr, as in standard FBA, to total (*lb*, *ub*, in mmol/hr):
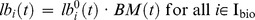
(5)

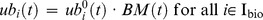
(6)


Next, in analogy with [13], we determine the minimal flux through a required metabolic functionality (RMF). Imposing RMF fluxes in TEAM is necessary in order to prevent the output of the trivial flux distribution ***V*** = 0. Because TEAM attempts to minimize the inconsistency between a flux distribution and gene expression data, the trivial solution is always optimal unless the user explicitly makes it infeasible. The only RMF used in this work is biomass production, although the TEAM protocol will work equally well for any other choice of RMF.

In this manuscript, measurements of the biomass flux were collected through growth data (*OD*(*t*)). When this is the case, TEAM can explicitly calculate the lower bound on the biomass flux to be
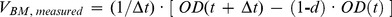
(7)where *d* is the death rate. In this work, we used *d* = 0.06.

The heart of the TEAM algorithm performs two optimization steps. In the first step, following [13], we minimize the inconsistency between metabolic flux and gene expression data. To do so, we associate a penalty, *c_i_*(*t*), with every reaction *i* in the model (see Gene Penalty Calculation section of Materials and Methods). This penalty reflects our expectation, based on gene expression, that a reaction is “inactive,” i.e. that it is unlikely to carry flux. The total penalty is minimized by solving the linear programming problem:
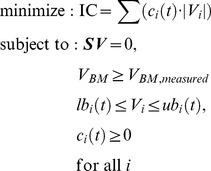
(8)Because there may be many alternative optimal solutions, we complete a secondary optimization to select the one that minimizes the sum of absolute value of all fluxes, while keeping the inconsistency constant. The minimization of the sum of absolute values of fluxes had been described before [30], and can be formulated as:
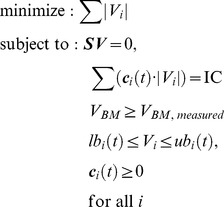
(9)


To complete the dFBA iteration, the media nutrient concentrations and the biomass are updated using the newly calculated exchange fluxes:
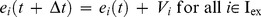
(10)

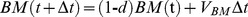
(11)The vector ***e*** is then used to assign lower bounds to all exchange fluxes in the next time step of TEAM using **Equation 4**.

### Gene Penalty Calculation

Gene penalties are used in the main optimization step of TEAM to identify flux states that minimize the flux through reactions with relatively low expression. As described in detail below, gene penalties are determined by comparing the expression value of a gene with a predefined threshold. Gene penalty calculation is done in three steps: threshold determination, expression comparison, and gene-to-reaction conversion.

#### Threshold determination

To determine either global (*x_global_*) or gene-specific thresholds (*x_g_*), a cumulative distribution function (CDF) is calculated from the union of the set of microarray measurements taken from the M3D Database [17] (*M3D*) and our experimental time-course measurements (*EXP*). This is done prior to use in the TEAM algorithm. Using a percentile value (*θ*), we can then calculate the global expression threshold, i.e. a gene expression value *x_global_* such that *θ* percent of all the M3D and EXP values will be less than *x_global_*, which we also express briefly in the following format:
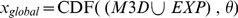
(12)The CDF in **Equation 12** is calculated using expression data from all genes for all experiments. In cases where we are concerned with a unique threshold for each gene *g*, we can use
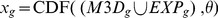
(13)where *M3D_g_* and *EXP_g_* correspond to the subsets of measurements pertaining to gene *g* from *M3D* and *EXP* respectively. Thus in **Equation 13**, the expression data used to generate the CDF only comes from measurements of gene *g*. For two examples of unique gene penalty calculation, see **Figure 3A–D**.

#### Penalty calculation

Once the gene threshold values have been calculated, the time-dependent penalties *p* are calculated during each iteration of TEAM using the experimental gene expression data that corresponds to the current time step. Three different penalty calculation methods were implemented: (Type 1, **Equation 14**) the global threshold method, (Type 2, **Equation 15**) the unique threshold method, and (Type 3, **Equation 16**) the unique threshold with standard deviation method:
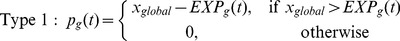
(14)

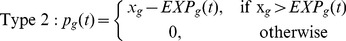
(15)

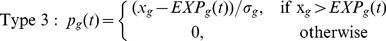
(16)


#### Gene-to-reaction conversion

In the simple case when one gene A encodes the enzyme responsible for a reaction *i* in the model, the penalty of reaction *i* (*c_i_*) is precisely equal to the penalty of gene A (*p_A_*). In general, however, since some reactions may be catalyzed by one or more (potentially multimeric) protein enzymes, gene penalties *p_i_* must be converted to reaction penalties *c_i_* in order to be compatible with the TEAM calculations. Conveniently, many stoichiometric models contain Boolean rules that define the relationship between genes and their corresponding reactions, making this evaluation possible. In Boolean gene-to-reaction mappings, an AND relation, such as “reaction 1 = gene A AND gene B,” describes a situation where gene A and gene B work together in an enzyme complex to catalyze reaction 1. In this case, we assume that the gene with the highest penalty is the one with the lowest expression relative to its corresponding threshold, and so will act to limit the flux through reaction 1. Our evaluation rule is therefore:
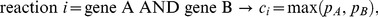
(17)where *c_i_* is the reaction penalty value and *p_A_* and *p_B_* are the gene penalty values calculated above for individual genes. OR relations such as “reaction 2 = gene A OR gene B” typically describe situations in which genes A and B act as isoenzymes that catalyze the same reaction. Because flux through reaction 2 can travel only as fast as the enzyme that is most active, we choose the minimum penalty value to represent the reaction penalty:
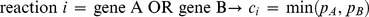
(18)


### Experimental Data and Metabolic Model

We applied TEAM to data obtained from a growth experiment of *S. oneidensis* MR-1 in carbon-limited conditions, as described by [18]. The data set contained three types of data: a time-series gene expression data set, a time-series media metabolite concentration data set, and a time-series measurement of biomass. The gene expression data was measured using an *S. oneidensis* MR-1 microarray Affymetrix chip platform and included 19 measurements at various time points between 0 and 50 hours. To derive the background data set used to calculate gene-specific penalty thresholds, we combined this set of 19 microarrays with 262 compatible microarray data sets obtained from the M3D database [17]. All gene expression data was normalized using the “affyrma” function in the Bioinformatics Toolbox in MATLAB [31]. The external metabolite concentration data set was measured using high performance liquid chromatography (HPLC), and provided an abundance profile for various metabolites in the media over time. This data set was not integrated directly using TEAM, but was used to measure the performance of our method against experimental data, as well as to define our starting media condition. Finally, the biomass growth dataset was measured using optical density (OD). This data was used in conjunction with a genome-scale metabolic model iSO783 of *S. oneidensis* MR-1 described in [21]. This model contains 774 reactions encoded by 783 genes, and 634 unique metabolites. The model includes the gene-to-reaction mapping used to associate gene information with the reactions in the metabolic model, as described above.

### Validation of TEAM with Supplementary Data

#### Data for yeast upon shift from fermentative to glycerol-based metabolism

Gene expression data was obtained from the Gene Expression Omnibus for the experiment from [26]. Samples from all ten time points were used to assemble a small compendium of gene expression measurements for each gene. To run TEAM, the yeast model iMM904 [32] was used in conjunction with the glycerol-based media described in [26]. Since no data was provided regarding the growth rate of yeast in this experiment, we instead elected to calculate the maximum possible biomass flux and then enforce that the metabolic model produce at least a minimum percentage *p* of this maximum. The results in **Figure S4** illustrate the results for *p* = 0.5, but the results generically hold for all *p* that we tested.

We compared the performance of Type 1 (global) and Type 2 (gene-specific) thresholds by completing a single iteration of TEAM for each possible penalty percentile *θ*, from 1 to 99%. For each *θ*, the resulting inconsistency score (IS) was obtained and normalized by the average penalty across all genes. Then, the inconsistency scores for the Type 1 and Type 2 methods were compared, shown in **Figure S4**. The “step-like” IS scores for the Type 2 method result from the very small compendium of gene expression measurements available to us (ten). Thus, at discrete percentiles, the penalty threshold for each gene in the model simultaneously changes, resulting in discrete changes in the IS.

#### Data on yeast metabolic oscillations

Gene expression data was obtained from [27]. Samples from all thirty six time points were again used to construct a compendium of gene expression measurements for each gene. The yeast model iMM904 was used in tandem with a media composition corresponding to the one described in [27]. We used gene expression data corresponding to hour 11 in the metabolic cycle, when the population of yeast secreted acetate and ethanol into the medium. As before, no biomass data was provided, so we instead chose to calculate the maximum possible biomass flux and then enforce that the metabolic model produce at least a minimum percentage *p* of this maximum. The results in **Figure S5** illustrate the results for *p* = 0.5, but the results generically hold for all *p* that we tested. We then compared the number of percentile thresholds *θ* (for which Type 1 (global) and Type 2 (gene-specific) thresholding predicted the secretion of acetate and ethanol. The results are shown in **Figure S5**. We also repeated the inconsistency score analysis described in the prior subsection.

## Supporting Information

Dataset S1 **Predictions of time-dependent metabolic fluxes for Type 1, 2, and 3 thresholding methods across three different percentiles** ***θ*** **= 65%, 72%, and 85%.** There are a total of nine different Excel files, compressed into a single zipped directory, which contains also a README file.
(ZIP)

Figure S1 **Sensitivity analysis for Type 1 global threshold.** (A) Total carbon concentration in media for each penalty threshold *θ*, summed over all time points. Acetate is the only intermediate carbon source found in the media over all penalty thresholds. (B) Extinction time of lactate and ammonium in the media. Lactate runs out earlier than ammonium for all penalty thresholds. Heatmap indicates the total media concentration of secreted carbon sources (acetate, pyruvate, glycolate, formate).
(TIF)

Figure S2 **Sensitivity analysis for Type 3 gene-specific threshold normalized by standard deviation.** Results are very similar to those in **Figure 4**. (A) Total carbon concentration in media for each penalty threshold *θ*, summed over all time points. Penalty thresholds between 40% and 75% exhibit enrichment for secreted carbon sources formate, glycolate, pyruvate. (B) Extinction time of lactate and ammonium in the media. Lactate runs out significantly earlier for intermediate penalty thresholds. Heatmap indicates the total media concentration of secreted carbon sources (acetate, pyruvate, glycolate, formate).
(TIF)

Figure S3 **Central carbon metabolism of** ***S. oneidensis*** **.** For a more detailed description of the reactions and metabolites, refer to **Tables S1** and **S2**.
(TIF)

Figure S4 **A comparison of overall inconsistency between Type 1 and Type 2 cutoffs for yeast grown on various media.** Data analyzed here was taken from [11]. For penalty thresholds *θ* between 1% and 99%, the total inconsistency score (IS) between gene expression and flux was measured and normalized by the average inconsistency among all fluxes. The blue line represents the IS using a global threshold (Type 1), and the green line represents the IS using a gene-specific threshold (Type 2). For all *θ*, the gene-specific threshold produces a flux distribution that is more consistent with the gene expression data. Percentiles *θ* which trivially produced flux distributions with no penalized reactions (resulting in an IS of zero) are not plotted.
(TIF)

Figure S5 **A comparison of overall inconsistency between Type 1 and Type 2 cutoffs for yeast undergoing metabolic cycles.** Data analyzed here was taken from [27]. (A) Occurrence of acetate excretion in the external media for both global (Type 1, blue) and gene-specific (Type2, green) thresholds. Flux solutions using gene-specific threshold produced roughly twice as many correct predictions of acetate production as compared to solutions using a global cutoff. (B) Total inconsistency score between gene expression and flux for penalty thresholds *θ* between 1% and 99%. Missing values correspond to an IS value of zero. Percentiles *θ* which trivially produced flux distributions with no penalized reactions (resulting in an IS of zero) are not plotted.
(TIF)

Table S1 **Reactions in central carbon metabolism of** ***S. oneidensis*** **.**
(DOC)

Table S2 **Metabolites in central carbon metabolism of** ***S. oneidensis*** **.**
(DOC)
