# Supplementary figures and images for: Temporal Expression-based Analysis of Metabolism
Source: PLoS Comput Biol. 2012 Nov 29;8(11):e1002781. doi: 10.1371/journal.pcbi.1002781 (PMC3510039; doi:10.1371/journal.pcbi.1002781)

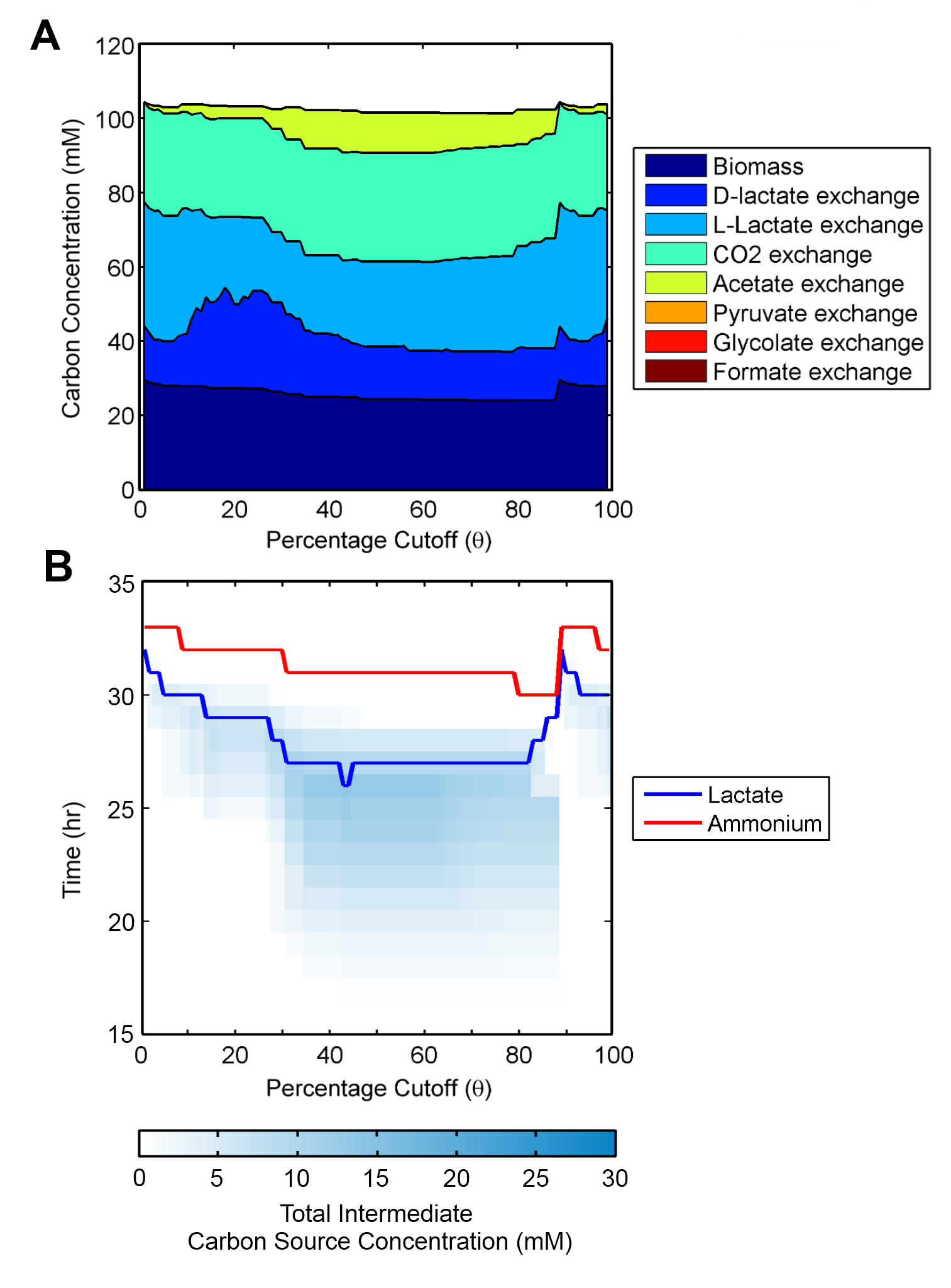

Supplement: Figure S1 — Sensitivity analysis for Type 1 global threshold. (A) Total carbon concentration in media for each penalty threshold θ, summed over all time points. Acetate is the only intermediate carbon source found in the media over all penalty thresholds. (B) Extinction time of lactate and ammonium in the media. Lactate runs out earlier than ammonium for all penalty thresholds. Heatmap indicates the total media concentration of secreted carbon sources (acetate, pyruvate, glycolate, formate). (TIF) [file pcbi.1002781.s002.tif]

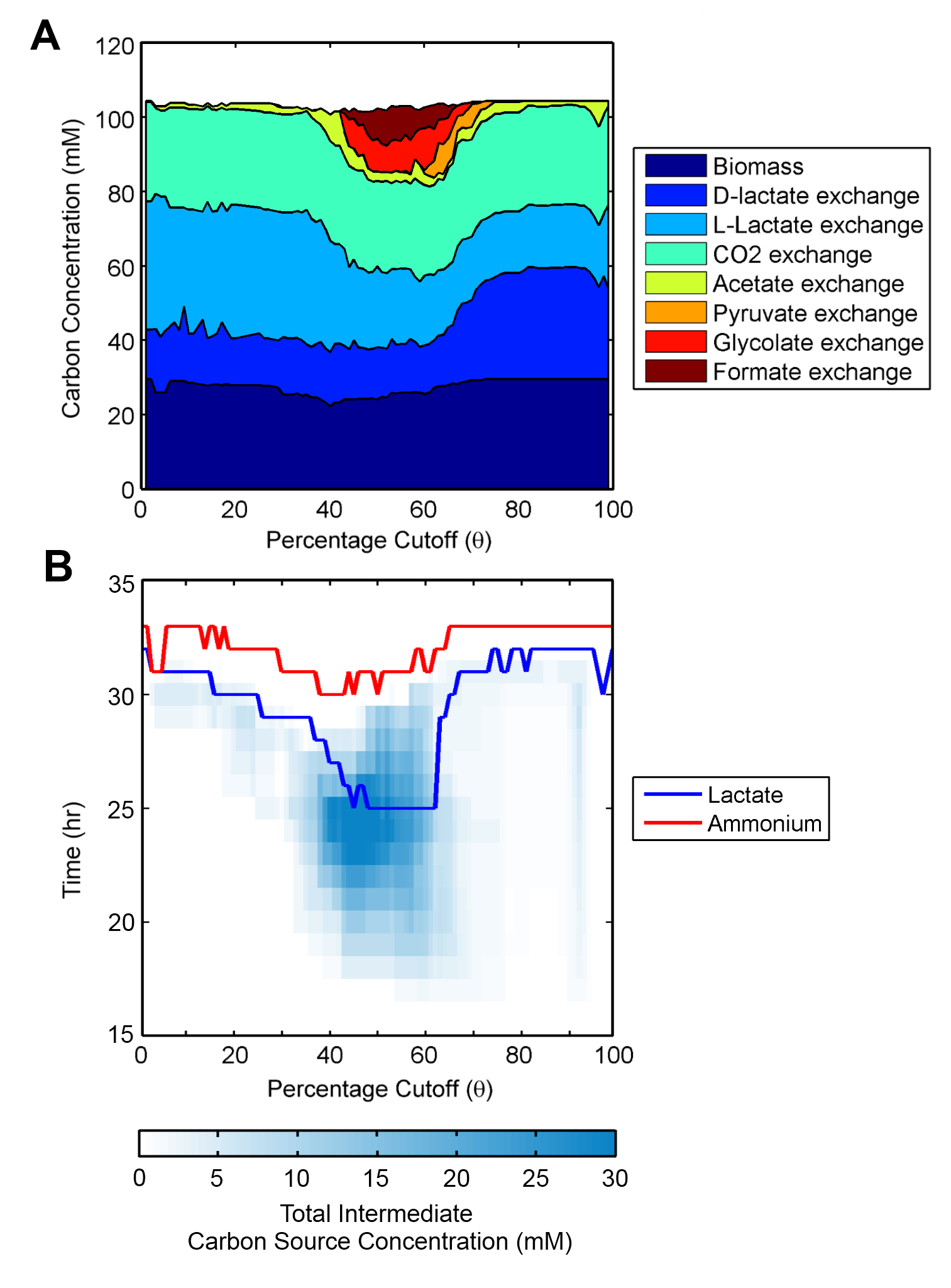

Supplement: Figure S2 — Sensitivity analysis for Type 3 gene-specific threshold normalized by standard deviation. Results are very similar to those in Figure 4 . (A) Total carbon concentration in media for each penalty threshold θ, summed over all time points. Penalty thresholds between 40% and 75% exhibit enrichment for secreted carbon sources formate, glycolate, pyruvate. (B) Extinction time of lactate and ammonium in the media. Lactate runs out significantly earlier for intermediate penalty thresholds. Heatmap indicates the total media concentration of secreted carbon sources (acetate, pyruvate, glycolate, formate). (TIF) [file pcbi.1002781.s003.tif]

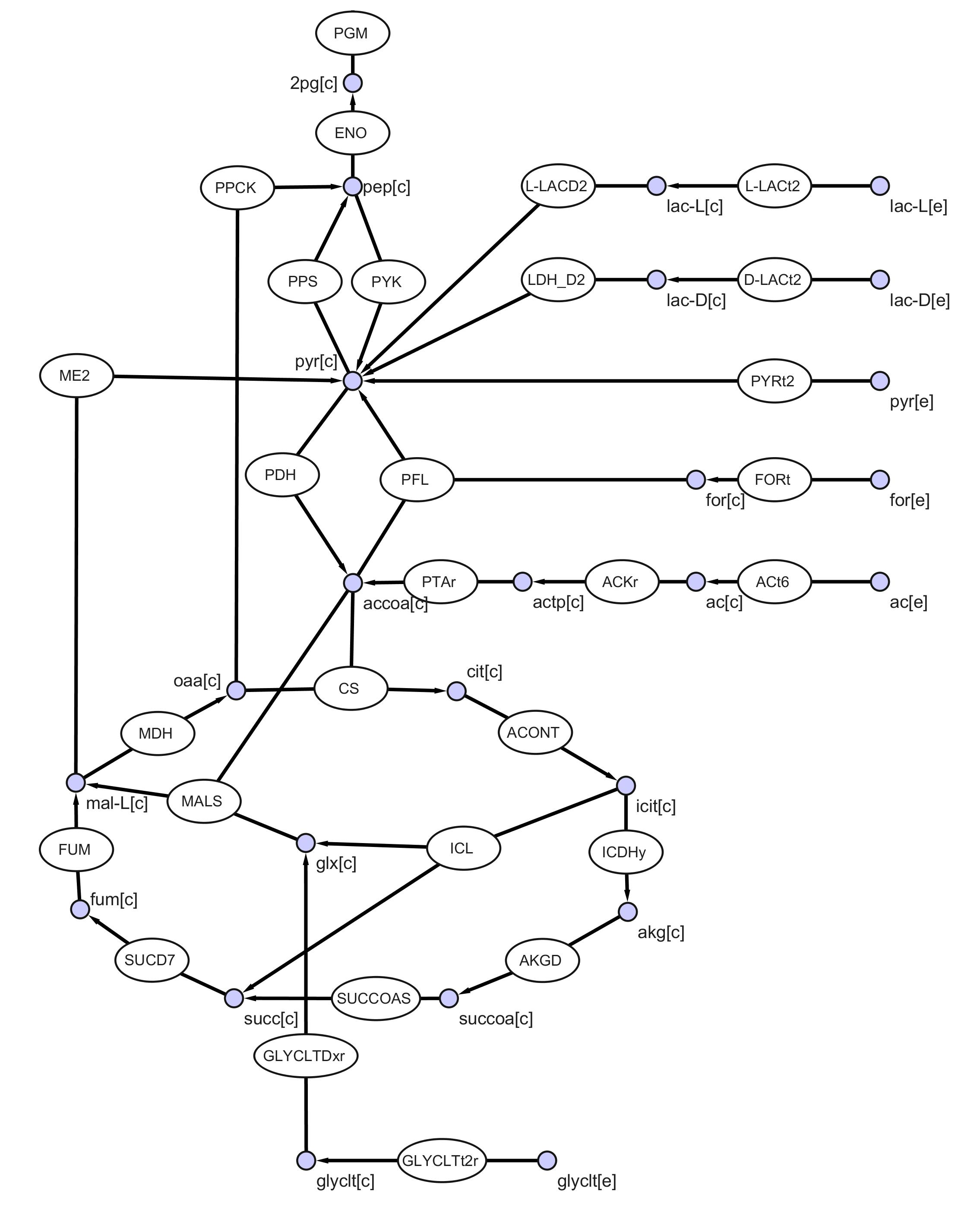

Supplement: Figure S3 — Central carbon metabolism of S. oneidensis . For a more detailed description of the reactions and metabolites, refer to Tables S1 and S2. (TIF) [file pcbi.1002781.s004.tif]

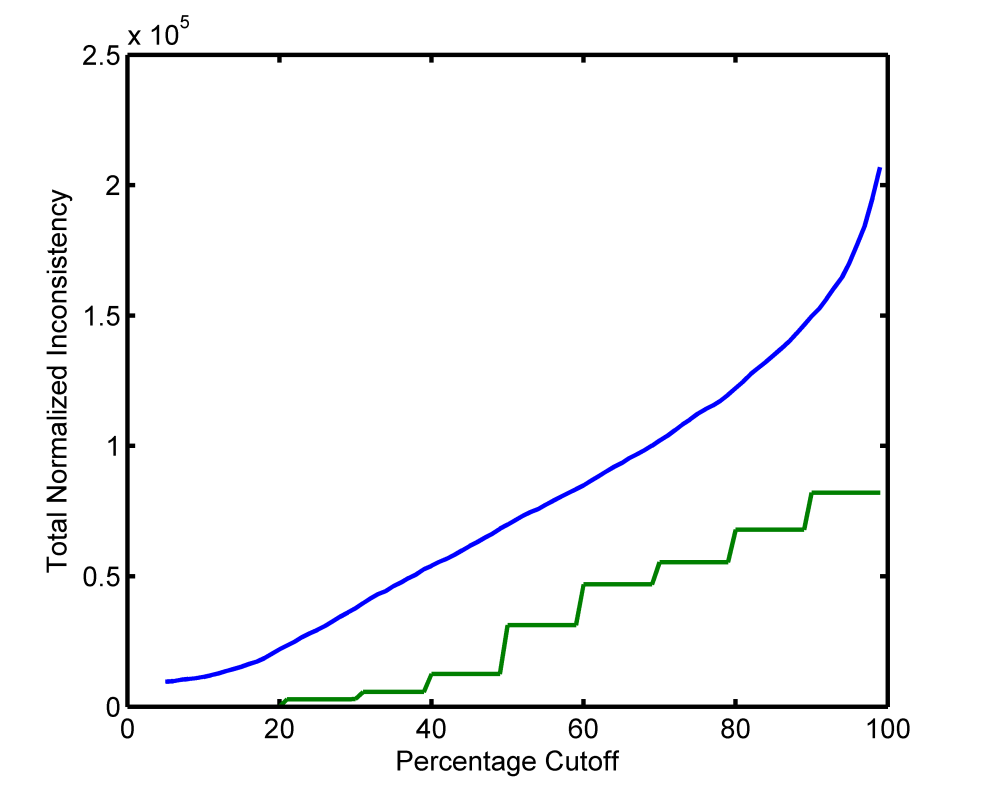

Supplement: Figure S4 — A comparison of overall inconsistency between Type 1 and Type 2 cutoffs for yeast grown on various media. Data analyzed here was taken from [11]. For penalty thresholds θ between 1% and 99%, the total inconsistency score (IS) between gene expression and flux was measured and normalized by the average inconsistency among all fluxes. The blue line represents the IS using a global threshold (Type 1), and the green line represents the IS using a gene-specific threshold (Type 2). For all θ, the gene-specific threshold produces a flux distribution that is more consistent with the gene expression data. Percentiles θ which trivially produced flux distributions with no penalized reactions (resulting in an IS of zero) are not plotted. (TIF) [file pcbi.1002781.s005.tif]

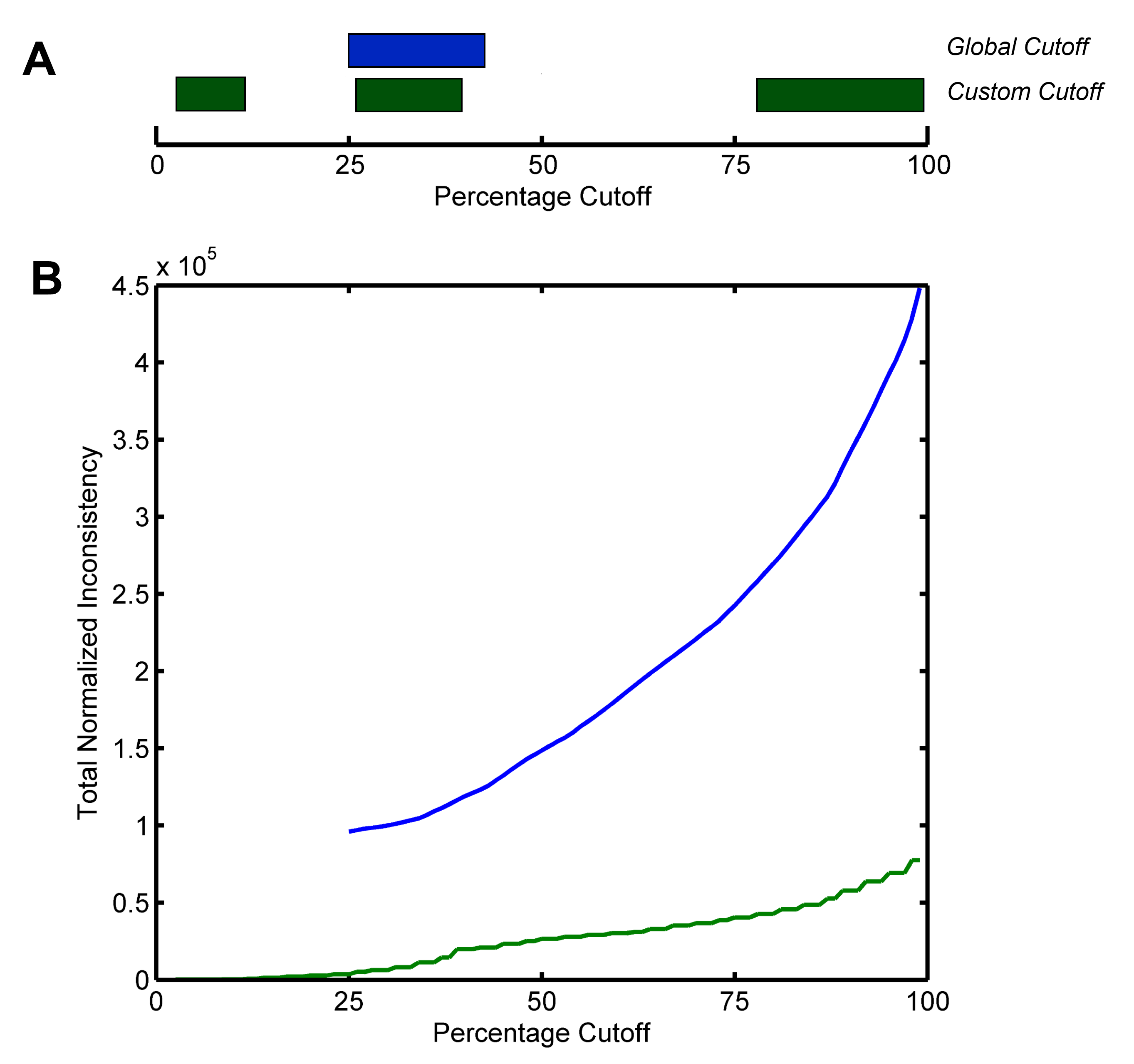

Supplement: Figure S5 — A comparison of overall inconsistency between Type 1 and Type 2 cutoffs for yeast undergoing metabolic cycles. Data analyzed here was taken from [27]. (A) Occurrence of acetate excretion in the external media for both global (Type 1, blue) and gene-specific (Type2, green) thresholds. Flux solutions using gene-specific threshold produced roughly twice as many correct predictions of acetate production as compared to solutions using a global cutoff. (B) Total inconsistency score between gene expression and flux for penalty thresholds θ between 1% and 99%. Missing values correspond to an IS value of zero. Percentiles θ which trivially produced flux distributions with no penalized reactions (resulting in an IS of zero) are not plotted. (TIF) [file pcbi.1002781.s006.tif]
