# Supplementary material for: Temporal Expression-based Analysis of Metabolism
Source: PLoS Comput Biol. 2012 Nov 29;8(11):e1002781. doi: 10.1371/journal.pcbi.1002781 (PMC3510039; doi:10.1371/journal.pcbi.1002781)
Supplement: Table S1 — Reactions in central carbon metabolism of S. oneidensis . (DOC) [file pcbi.1002781.s007.doc]

| **Symbol** | **Reaction Name** | **Equation** |
| --- | --- | --- |
| ACKr | Acetate Kinase | [c] : ac + atp <==> actp + adp |
| ACONT | Aconitase | [c] : cit <==> icit |
| ACt6 | Acetate transport | ac[e] + h[e] <==> ac[c] + h[c] |
| AKGD | 2-Oxoglutarate Dehydrogenase | [c] : akg + coa + nad --> co2 + nadh + succoa |
| CS | Citrate Synthase | [c] : accoa + h2o + oaa --> cit + coa + h |
| D-LACt2 | D-lactate transport | h[e] + lac-D[e] <==> h[c] + lac-D[c] |
| ENO | Enolase | [c] : 2pg <==> h2o + pep |
| FORt | Formate transport | for[e] <==> for[c] |
| FUM | Fumarase | [c] : fum + h2o <==> mal-L |
| GLYCLTDxr | Glycolate Dehydrogenase (NAD) | [c] : glyclt + nad <==> glx + h + nadh |
| GLYCLTt2r | Glycolate transport | glyclt[e] + h[e] <==> glyclt[c] + h[c] |
| ICDHy | Isocitrate Dehydrogenase (NADP) | [c] : icit + nadp <==> akg + co2 + nadph |
| ICL | Isocitrate Lyase | [c] : icit --> glx + succ |
| LDH_D2 | D-lactate dehydrogenase | [c] : lac-D + ubq8 --> pyr + ubq8h2 |
| L-LACD2 | L-lactate dehydrogenase | [c] : lac-L + ubq8 --> pyr + ubq8h2 |
| L-LACt2 | L-lactate transport | h[e] + lac-L[e] <==> h[c] + lac-L[c] |
| MALS | Malate Synthase | [c] : accoa + glx + h2o --> coa + h + mal-L |
| MDH | Malate Dehydrogenase | [c] : mal-L + nad <==> h + nadh + oaa |
| ME2 | Malic Enzyme (NADP) | [c] : mal-L + nadp --> co2 + nadph + pyr |
| PDH | Pyruvate Dehydrogenase | [c] : coa + nad + pyr --> accoa + co2 + nadh |
| PFL | Formate C-acetyltransferase | [c] : coa + pyr <==> accoa + for |
| PPCK | Phosphoenolpyruvate carboxykinase | [c] : atp + oaa --> adp + co2 + pep |
| PPS | Phosphoenolpyruvate synthase | [c] : atp + h2o + pyr --> amp + (2) h + pep + pi |
| PTAr | Phosphotransacetylase | [c] : accoa + pi <==> actp + coa |
| PYK | Pyruvate Kinase | [c] : adp + h + pep --> atp + pyr |
| PYRt2 | Pyruvate transport | h[e] + pyr[e] <==> h[c] + pyr[c] |
| SUCD7 | Succinate Dehydrogenase | [c] : succ + ubq8 --> fum + ubq8h2 |
| SUCOAS | Succinyl-CoA Synthetase | [c] : atp + coa + succ <==> adp + pi + succoa |

**Table S1**. Reactions corresponding with network diagram of internal fluxes. Data presented below was adapted from the *S. oneidensis*  metabolic model iSO783.
