# Supplementary material for: Temporal Expression-based Analysis of Metabolism
Source: PLoS Comput Biol. 2012 Nov 29;8(11):e1002781. doi: 10.1371/journal.pcbi.1002781 (PMC3510039; doi:10.1371/journal.pcbi.1002781)
Supplement: Table S2 — Metabolites in central carbon metabolism of S. oneidensis . (DOC) [file pcbi.1002781.s008.doc]

**Table S2**. Metabolites corresponding with network diagram of internal fluxes. Data presented below was adapted from the *S. oneidensis*  metabolic model iSO783.

| **Symbol** | **Metabolite Name** |
| --- | --- |
| 2pg[c] | D-Glycerate 2-phosphate |
| ac[c] | Acetate |
| ac[e] | Acetate, external |
| accoa[c] | Acetyl-CoA |
| actp[c] | Acetyl phosphate |
| akg[c] | 2-Oxoglutarate |
| cit[c] | Citrate |
| for[c] | Formate |
| for[e] | Formate, external |
| fum[c] | Fumarate |
| glx[c] | Glyoxylate |
| glyclt[c] | Glycolate |
| glyclt[e] | Glycolate, external |
| icit[c] | Isocitrate |
| lac-D[c] | D-Lactate |
| lac-D[e] | D-lactate, external |
| lac-L[c] | L-Lactate |
| lac-L[e] | L-lactate, external |
| mal-L[c] | L-Malate |
| oaa[c] | Oxaloacetate |
| pep[c] | Phosphoenolpyruvate |
| pyr[c] | Pyruvate |
| pyr[e] | Pyruvate, external |
| succ[c] | Succinate |
| succoa[c] | Succinyl-CoA |
